# Postoperative pain and pain management and neurocognitive outcomes after non-cardiac surgery: a protocol for a series of systematic reviews

**DOI:** 10.1186/s13643-022-02156-3

**Published:** 2022-12-24

**Authors:** Maram Khaled, Denise Sabac, Maura Marcucci

**Affiliations:** 1 Department of Health Research Methods, Evidence and Impact, McMaster University, 1280 Main Street W, Hamilton, ON L8S4L8 Canada; 2 Perioperative and Surgery Research Program, Population Health Research Institute, Hamilton, ON Canada; 3 Bachelor of Health Sciences, McMaster University, Hamilton, ON Canada; 4 Department of Medicine, McMaster University, Hamilton, ON Canada

**Keywords:** Surgery, Delirium, Cognitive dysfunction, Pain, Opioid, Analgesia, Pain management

## Abstract

**Background:**

Postoperative delirium (POD) is common after non-cardiac surgery in older adults and can result in increased risk of adverse outcomes including postoperative cognitive dysfunction (POCD). Pain after surgery is also frequent and can persist as chronic postsurgical pain (CPSP). Evidence is inconsistent and controversial on whether acute and chronic postsurgical pain, and different postoperative pain management strategies (including opioid versus opioid-sparing strategies), is associated with the occurrence of POD and POCD. In this protocol, we propose a series of systematic reviews to answer the following research questions: In adults undergoing non-cardiac surgery, (1) is acute postsurgical pain associated with POD and/or POCD? (2) Are opioid-sparing/avoidance strategies of acute postoperative pain management associated with lower incidence and/or severity of POD and POCD, compared to predominantly opioid-based strategies? (3) Is CPSP associated with POCD? (4) Are opioid-sparing management strategies of CPSP associated with lower incidence and/or severity of POCD compared to standard of care or strategies not aiming at reduced opioid use?

**Methods:**

We will search MEDLINE, EMBASE, Cochrane (CENTRAL), CINAHL, and PSYCHINFO. According to the research question, we will include cohort and case-control studies (questions 1 and 3) or randomized controlled trials and non-randomized studies (questions 2 and 4). The risk of bias will be assessed independently and in duplicate using the revised Cochrane risk-of-bias tool, the Newcastle-Ottawa Scale, and the Joanna-Briggs Institute critical appraisal checklist. Disagreements will be resolved by a third reviewer. Findings will be reported narratively, and where possible and appropriate, meta-analyses will be performed. Certainty of evidence will be assessed using the Grading of Recommendations Assessment, Development, and Evaluation approach. We will conduct the reviews in accordance with the guideline of the Preferred Reporting Items for Systematic Review and Meta-Analyses Protocols.

**Discussion:**

Our systematic reviews will summarize available evidence to date on the association of postoperative pain and its management strategies with the incidence of POD and POCD in non-cardiac surgery. We will evaluate the existing evidence and its limitations and inform the design of future interventional studies comparing the effects of different pain management strategies on postoperative neurocognitive outcomes.

**Systematic review registration:**

PROSPERO CRD42021192105

**Supplementary Information:**

The online version contains supplementary material available at 10.1186/s13643-022-02156-3.

## Background

### Introduction

Delirium is an acute state of confusion characterized by altered attention, disorientation, memory deficit, language deficit, and impaired perception [1]. Surgery can be a trigger of delirium with a reported incidence of postoperative delirium (POD) ranging between 5 and 50% [2]. Although a higher incidence of POD is reported after cardiac surgery, the incidence of POD after non-cardiac surgery is also high (7–26%) [2, 3]. POD is more common in older adults, and in particular those with multiple comorbidities and polypharmacy [2]. POD is associated with an increased risk of adverse outcomes, including prolonged hospital stay, higher re-admission rates, increased need for one-to-one supervision, and increased mortality [4].

Surgery has been also associated with a short- and long-term decline in cognitive performance. Postoperative cognitive dysfunction (POCD) has been defined as a condition characterized by cognitive impairment after surgery as compared to the preoperative performance, detected up to 1 year after surgery [5]. The reported incidence of POCD after non-cardiac surgery varies in the literature because of the heterogeneity in instruments and timing for assessment [6], but recent data in mixed non-cardiac surgical populations 65 years or older suggest it can be as high as 30% [7, 8]. Patients who experience POD have been found to be at higher risk of POCD [3], but POCD can occur even in the absence of POD.

### Rationale

The etiology and pathogenesis of POD and POCD are not fully understood. Drug-induced delirium has been reported, especially with medications associated with anticholinergic-like effects [9]. Opioids have been associated with an increased risk of POD [10–12]. On the other side, undertreated acute pain is also a predisposing factor for delirium [11]. Moreover, many patients experience pain for a long time after surgery [13]. Chronic postsurgical pain (CPSP) occurs as a result of a cascade of events, initiated by surgical trauma, including neurotransmitter release and peripheral and central sensitization [14]. Pain sensation is related to perception and cognition [15]. There is evidence to suggest an association of persistent inflammation and pain with impaired cognition after surgery [16–18]. A bidirectional interplay between chronic pain and cognitive dysfunction has been suggested [19]. In a longitudinal community-based study, a history of persistent pain (pain of any origin lasting for at least 2 years) at baseline was associated with memory decline, and even dementia, over 10 years of follow-up [20].

To our knowledge, so far, two systematic reviews have been conducted to compile evidence on perioperative analgesia and POD and POCD [21, 22]; one was published in 2006 (search date 2005), and one in 2017 (search date 2014). Both reviews mainly focused on comparing the effect of different perioperative opioids. Recently, more studies have been conducted to assess the effect of analgesic modalities aimed at minimizing perioperative opioid use and eventually possible opioid-related complications [23–28]. Moreover, some of the studies included in the two systematic reviews intended to look at the effect of perioperative analgesia on “POCD”; however, they only assessed participants in the immediate postoperative period. Only more recently has the concept of POCD, as distinct from POD and as a condition possibly persisting for months after surgery, been better defined [5]. Finally, no systematic review has so far summarized the existing evidence on the association of CPSP and its treatment, with persistent POCD.

### Objectives

We are conducting a series of four systematic reviews to summarize and appraise the cumulative evidence on perioperative pain and pain management and the incidence of POD and POCD in adult patients undergoing or who underwent non-cardiac surgery, in the short and long term. In particular, we aim to answer the following questions (Table 1):

1. Is acute postsurgical pain associated with POD (incidence and severity) and/or POCD?
2. Are opioid-sparing or avoidance pharmacological and non-pharmacological management strategies of acute postoperative pain associated with lower incidence and/or severity of POD and POCD, compared to predominantly opioid-based strategies?
3. Is CPSP associated with POCD?
4. Are opioid-sparing pharmacological and non-pharmacological management strategies of CPSP associated with lower incidence and/or severity of POCD compared to standard of care or strategies not aiming at reduced opioid use?

**Table 1:** Research questions according to the PICO format

Research question	Population	Intervention/exposure and comparators	Outcomes
Q1	Adults undergoing non-cardiac surgery	Exposure: acute (presence) or moderate to severe (intensity) postsurgical pain Comparator: no or mild acute postsurgical pain	POD or POCD
Q2	Adults undergoing non-cardiac surgery	Intervention: pharmacological and non-pharmacological management strategies to treat or prevent perioperative acute pain with proved or intended opioid-sparing effect Comparator: pain management strategy without proved or intended opioid-sparing effect or standard of care	Primary: POD or POCD Secondary: perioperative acute pain, CPSP
Q3	Adults undergoing non-cardiac surgery	Exposure: CPSP (presence or intensity) Comparator: no CPSP or less intense CPSP	POCD
Q4	Adults undergoing non-cardiac surgery	Intervention: pharmacological and non-pharmacological pain management strategies to treat or prevent PPSP with proved or intended opioid-sparing effect Comparator: pain management strategy without proved or intended opioid-sparing effect or standard of care	Primary: POCD Secondary: CPSP

Refer to Table 1 for the research questions according to the PICO format.

## Methods

We have prepared this protocol for our systematic reviews in accordance with the guideline of the Preferred Reporting Items for Systematic Review and Meta-Analyses-Protocols (PRISMA-P). A populated PRISMA-P checklist is provided (see Additional file 1).

### Study selection

We will include studies in any language if they evaluate the aforementioned research questions and meet the following criteria.

#### Population

Adults undergoing elective or non-elective non-cardiac surgery, excluding cranial neurosurgery. We will include all studies in adults (i.e., ≥ 18 years old) but plan to report and summarize the evidence on the older population (i.e., ≥ 65 years old) whenever possible.

#### Intervention/exposure and comparator

For questions Q1 and Q3 (Table 1), studies will be included if they reported the incidence and/or severity of acute postsurgical pain and/or CPSP, measured by any scale including the visual analog scale, numeric pain rating scale, or brief pain inventory [29, 30]. We will consider acute postsurgical pain as pain occurring up to 7 days after surgery, and CPSP as “chronic pain that develops or increases in intensity after a surgical procedure and persists beyond the healing process, i.e., at least 3 months after the surgery” according to the definition of the International Association for the Study of Pain [31]. Studies reporting mild vs. moderate or severe postoperative pain without specifying the measurement tool will also be included. We will include studies evaluating chronic pain of different etiologies, as long as they specify that their population also included people who underwent surgery; we will extract relevant data on this subgroup whenever available. For questions Q2 and Q4 (Table 1), studies will be included if they examined pre-, intra-, or postoperative interventions for preventing or managing postsurgical pain, proven or intended to minimize opioid use (opioid-sparing), as compared to alternative interventions, or no active intervention/placebo/standard or usual care. This definition will comprise (1) pharmacological interventions, including different types or doses of opioids; (2) regional analgesic techniques including nerve blocks; and (3) non-pharmacological interventions, e.g., physiotherapy and psychotherapy [32]. We will consider single or multi-modal interventions.

#### Outcomes

Our primary outcomes of interest are POD (questions Q1 and Q2) and POCD (all questions). We expect to find variability in how different studies measured these outcomes. We will initially include studies regardless of the definition and instruments used, as long as the investigators intended to assess POD and POCD (i.e., based on face validity of their definitions). Secondarily, we will report and summarize the evidence only including studies in which (1) POD was defined in agreement with the Diagnostic and Statistical Manual for Mental Disorders, fifth edition (DSM-5) terminology and assessed by a validated tool, such as the Confusion Assessment Method [33] or the Memorial Delirium Assessment Scale [34], and (2) POCD was defined as a decline in cognitive function(s) occurring up to 12 months after surgery as compared to the preoperative performance, and as assessed by validated neuropsychological tests such as Montreal Cognitive Assessment [35]. Studies will be included whether POD and POCD are the primary outcomes in the eligible studies or not. For studies on interventions reporting results on our primary outcomes of interest, we will also collect data, when available, on the effects of those interventions on acute and persistent pain.

#### Study design

For Q1 and Q3, we will include cohort, case-control, and cross-sectional studies. For Q2 and Q4, we will include randomized controlled trials (RCTs) and non-randomized studies. We will not include cases series.

### Search strategy for identification of relevant studies

The following electronic databases will be searched from their inception to date: MEDLINE (1946–date), EMBASE (1974–date), Cochrane Central Register for Controlled Trials (CENTRAL), CINAHL, and PSYCHINFO. In addition, national and international clinical trial registries (e.g., WHO ICTRP and ClinicalTrials.gov) will be checked for ongoing trials and corresponding researchers will be contacted for their data if trials are completed but not published. We will also search gray literature by targeted google searches (first 100 hits) and databases like Health Canada and Open Grey. Simultaneous to the electronic search, the reference list of relevant studies and systematic reviews will be manually checked. Field experts will be contacted in person or via email for other references. The most important concepts from our review questions (population and intervention) were used to build a list of words selected by scanning relevant studies as well as databases for text words, controlled vocabulary, and mesh terms which were used in the search. Search terms for the neurocognitive outcome were added when the primary search returned an enormous amount of hits that were mostly irrelevant to the objective of the review. The search strategy was revised by an expert librarian. An example of the detailed search strategy, used for the MEDLINE database, is outlined in an additional file (see Additional file 2).

### Screening and data abstraction

Initial title and abstract screening will be done independently by pairs of reviewers. Any article that is clearly ineligible will be excluded at that stage. All full-text articles deemed eligible for inclusion from the previous step will then be independently screened by pairs of reviewers using specific eligibility criteria via a pretested screening form. Disagreements will be resolved by consensus or via third-reviewer adjudication if necessary. The number of articles included and excluded at the various stages will be documented.

Data abstraction will be conducted by pairs of reviewers, independently and in duplicate, using standardized pretested data extraction forms which will be piloted. The following information will be extracted: study characteristics (design, year, duration of follow-up, sample size per each study arm, setting, and country), participant characteristics (age, gender, type of surgery), intervention or exposure details (type of intervention, route of administration, dose, duration, frequency of administration, pain score, and opioid consumption), and results (risk statistics and their corresponding measures of variance for dichotomous outcomes and means and their corresponding measures of variance for continuous outcomes for each of the reported time-points). In the case of disagreement between pairs, reviewers will be asked to come to a consensus. If a consensus cannot be reached, a third reviewer will be consulted.

### Risk of bias assessment and assessment of the level of evidence

For randomized trials, the risk of bias will be assessed using the revised Cochrane risk-of-bias tool for randomized trials (RoB 2) [36]. The following five domains of bias will be assessed: the randomization process (including sequence generation and allocation concealment), deviations from intended interventions, missing outcome data, measurement of the outcome, and selective outcome reporting. The RoB 2 algorithm for suggested judgment of risk of bias will be used as a guide to determine the judgment for each domain. For interventional non-randomized trials, the Risk Of Bias In Non-randomized Studies–of Interventions (ROBINS-I) assessment tool will be used to assess the risk of bias [37]. The following seven domains will be assessed for bias: confounding, selection of participants, classification of interventions, deviations from intended interventions, missing data, outcome measurement, and selective reporting. The ROBINS-I guide for judgment will also be used to determine the overall risk of bias as mild, moderate, serious, or critical. For observational studies addressing our questions Q1 and Q3, we will use the Newcastle-Ottawa Scale (NOS) [38] for cohort and case-control studies and the Joanna Briggs Institute (JBI) critical appraisal checklist for analytical cross-sectional studies [39]. The risk of bias assessment will be done in duplicate for each outcome (two reviewers will independently assess each outcome).

The Grading of Recommendations Assessment, Development, and Evaluation (GRADE) approach will be used to assess the certainty of evidence for each of the prespecified outcomes. The five GRADE considerations (risk of bias, inconsistency, indirectness, imprecision, and publication bias) will be used to rate the quality as high, moderate, low, or very low. For RCTs, the evidence rating will be started as high and rated down if needed. For non-randomized trials, the level of evidence will be started at low and upgraded if there is a large effect, dose-response or plausible confounding opposing the effect. GRADE assessment will be done in duplicate for each outcome (2 reviewers will assess each outcome for GRADE). GRADEpro software will be used to prepare the summary of findings tables.

### Data synthesis and analysis

Statistical analyses will be conducted in RevMan (version 5.3). Meta-analyses will be performed when deemed appropriate based on the level of homogeneity in the design of the original studies. We will primarily perform random effects models. We will assess and report heterogeneity quantitatively using the *I*^2^ statistic and performing a chi-square test. *I*^2^ statistics will be interpreted according to guidelines from the Cochrane Handbook. For all meta-analyses with at least 10 studies, potential for publication bias will be visually assessed by funnel plot symmetry.

Q2 and Q4: For studies reporting the effects of interventions on dichotomous outcomes (i.e., incidence of POD and POCD), we will calculate and meta-analyze odds ratios (OR) with 95% confidence intervals (CIs). For studies reporting the effects of interventions on continuous outcomes (e.g., severity of POD, changes in cognitive scores), we will calculate and meta-analyze the pooled weighted mean difference (WMD) and/or standardized mean difference (SMD) with corresponding 95% CIs. In the decision of the feasibility of a meta-analysis and the meta-analytic methods to use, careful considerations will be given to (1) whether effect sizes are measured upon different scales and (2) whether treatment effects are measured and reported as post-intervention measurements or changes from baseline. For non-randomized studies, adjusted and non-adjusted effect sizes will be extracted (collecting information about the covariates included in the original analyses); adjusted measurements of effects will be prioritized for inclusion in our meta-analyses.

Q1 and Q3: We expect variability with which relevant observational studies might address our research questions, including looking at changes in the risk of POD or POCD (dichotomous outcome) per each unit of change in acute or chronic pain score; looking at the association between the risk of POD or POCD (dichotomous outcome) and the presence of acute or persistent pain according to a certain cut-off; and looking at the severity of POD or changes in cognitive scores per each unit of change in acute or chronic pain score, or associated with the presence of acute or persistent pain according to a certain cut-off. Also, we expect to find observational studies that purely looked at an association, including cross-sectional studies, and studies with a more appropriate design to infer about an exposure-outcome type of relationship. We will decide on whether and how to pool and meta-analyze studies accounting for this variability and based on the available data.

We plan to perform the following secondary analyses:

1. Meta-analyses including only RCTs for Q2 and Q4
2. Meta-analyses including only prospective cohort studies for Q1 and Q3
3. Meta-analyses including only studies with low risk of bias
4. Meta-analyses including only studies using standardized definitions and validated methods of assessment for our primary outcomes
5. Separate meta-analyses for studies evaluating POCD based on the time of assessment

## Discussion

The proposed set of reviews will systematically collect and appraise evidence coming from observational and interventional studies evaluating the association between perioperative pain and pain management strategies on the incidence of postoperative neurocognitive events, particularly POD and POCD. We will in particular look for studies comparing the effects of different opioids as well as the effect of opioids vs. opioid-sparing pain management strategies in the short and long term. This is important to understand as surgery is more frequently offered as a treatment option to older adults, who are also more susceptible to postsurgical neurocognitive complications. Given the suggested bidirectional relationship between pain and cognition and the association between opioids and POD, and possibly POCD, it is necessary to appraise available evidence, including the recent studies that were not evaluated in previous reviews. This will allow to establish whether the current cumulative evidence can already inform practice or, as we expect, has important limitations in quantity and quality and leaves gaps in knowledge that need to be addressed in future studies.

## Supplementary Information

**Additional file 1.** PRISMA-P Checklist.

**Additional file 2.** Search strategy for MEDLINE.

## Data Availability

Not applicable.

## Abbreviations

POCD: Postoperative cognitive dysfunction
POD: Postoperative delirium
CPSP: Chronic postsurgical pain
CI: Confidence interval
SMD: Standardized mean difference
DSM-5: Diagnostic and Statistical Manual for Mental Disorders, fifth edition
GRADE: Grading of Recommendations Assessment, Development, and Evaluation
PRISMA-P: Preferred Reporting Items for Systematic Review and Meta-analyses Protocols
CENTRAL: Cochrane Central Register for Controlled Trials
