# Supplementary material for: Postoperative pain and pain management and neurocognitive outcomes after non-cardiac surgery: a protocol for a series of systematic reviews
Source: Syst Rev. 2022 Dec 24;11:280. doi: 10.1186/s13643-022-02156-3 (PMC9789645; doi:10.1186/s13643-022-02156-3)
Supplement: Supplementary file 2 — Additional file 2. Search strategy for MEDLINE. [file 13643_2022_2156_MOESM2_ESM.docx]

Search Strategy for Medline

| [(surg* or operat* or postoperat* or perioperat*).tw., or exp Surgical Procedures, Operative/, or surg*.fs.], and [pain.tw., or pain/ or exp pain, postoperative/, or pain manag*.tw., or exp Pain Management, or exp Analgesics/, or analges*.tw., or opioid.tw., or analgesia/ or exp acupuncture analgesia/ or exp analgesia, epidural/ or exp analgesia, patient-controlled/ or exp neuroleptanalgesia/, or exp Nerve Block/, or block.tw., or exp Mind-Body Therapies/, or mind-body therap*.tw., or psychotherapy/ or exp cognitive behavioral therapy/ or exp relaxation therapy/, or exp gabapentin/ or exp pregabalin/, (dexmetomidine or dexmedetomidine).mp., or exp Dexmedetomidine/], and [(delirium or cogn*).tw., or exp Delirium/, or exp Cognitive Dysfunction/, or confusion assessment method.mp. , or delirium rating scale.mp., , or memorial delirium assessment scale.mp. , or (4As test or 4 a`s test).mp. , or General Practitioner Assessment of Cognition.mp. , or mini mental state examination.mp. , or "Mental Status and Dementia Tests"/, or Neuropsychological Tests/, or montreal cognitive assessment.mp.]. |
| --- |
